# Radiographic Evaluation of Syndesmosis Stabilisation Using the TightRope System Versus Syndesmotic Screw Fixation for the Management of Ankle Fractures With a Syndesmotic Injury

**DOI:** 10.7759/cureus.45910

**Published:** 2023-09-25

**Authors:** Georgios Saraglis, Anwar Khan, Harsh Chaudhari, Sagar Pyakurel, Sayed Fazal Elahi Rabbani, Mohamed Arafa

**Affiliations:** 1 Department of Trauma and Orthopaedics, Bedfordshire NHS Foundation Trust, Bedford, GBR; 2 Department of Trauma and Orthopaedics, Bedfordshire NHS Foundation Trust, Luton, GBR

**Keywords:** foot and ankle trauma, foot & ankle surgery, syndesmosis injury, tightrope fixation, ankle fracture management

## Abstract

Background

Ankle syndesmotic injuries represent complex orthopaedic injuries, commonly requiring open reduction and fixation. Several techniques have been described for fixation, with syndesmotic screw fixation being traditionally considered as the ‘gold standard’. Among the relatively new techniques developed, the TightRope system stabilisation provides ‘dynamic’ stabilisation with promising results. We aimed to evaluate the radiographic performance of these two different surgical techniques in the management of ankle fractures with an underlying syndesmotic injury.

Methods

A total of 85 cases were included in the study and were divided into two groups: syndesmotic screw fixation (48 cases) and TightRope system (37 cases). Patient demographics, type of ankle fracture and type of implant used were recorded for all the cases, and evaluation of the postoperative radiographs was performed for all. For all patients, the radiographic parameters assessed included the medial clear joint space (MCS), tibiofibular overlap (TFO), and anterior and posterior tibiofibular interval in order to calculate the anterior tibiofibular ratio (ATFR).

Results

Statistical analysis revealed no statistically significant differences in the radiographic parameters of the postoperative radiographs between the two groups. However, in the syndesmotic screw group, a higher incidence of radiographic malreduction was seen, as indicated by the MCS and ATFR parameters, in comparison to the TightRope fixation group. An equal distribution of radiographic abnormal parameters was noted among the different types of ankle fractures included in the study (trimalleolar, bimalleolar and isolated fibula fractures with syndesmotic injury) with no obvious positive correlation noted (Pearson correlation test).

Conclusion

Both surgical techniques seem to provide adequate reduction of the syndesmosis, with no statistical significant differences detected from the radiographic evaluation of both groups. In our study though, the syndesmotic screw group was associated with a higher incidence of radiographic malreduction as indicated by the MCS and ATFR parameters. The TightRope system seems to have a lower rate of radiographic malreduction and provides an equally effective way of syndesmosis fixation based on a dynamic mode of stabilisation.

## Introduction

Ankle fractures are among the most common orthopaedic injuries and frequently present with syndesmosis disruption, requiring a careful clinical assessment and preoperative planning [1]. The syndesmosis is a multi-ligament complex including the interosseous ligament, the inferior transverse tibiofibular ligament, and the anterior and posterior inferior tibiofibular ligament [2]; it is considered a key element with regard to the stability of the distal tibiofibular joint.

The diagnosis of a syndesmotic injury can be challenging, and many different methods have been described for the accurate diagnosis of syndesmotic disruption [3]. Computed tomography (CT) has been commonly used, in order to assess the rotational alignment or disruption of the syndesmosis, whereas magnetic resonance imaging (MRI) is highly sensitive in recognising and evaluating ligament disruption [4,5]. Ankle diagnostic arthroscopy has also been described as another alternative in diagnosing ankle syndesmotic injuries [6]; however, none of the above can easily be widely used and their economic effectiveness is strongly debatable [6].

Over the last few years, new radiographic parameters focused on the assessment of orthogonal views (anteroposterior, or AP, mortise view and true lateral view of the ankle) have been introduced [7], increasing the reliability and sensitivity in diagnosing syndesmotic injuries of the ankle based on simple orthogonal radiographs.

These radiographic parameters include two parameters while assessing the true AP radiograph of the ankle, the medial clear joint space (MCS) and the tibiofibular overlap (TFO), and two parameters while assessing the true lateral radiograph of the ankle, the anterior tibiofibular interval (ATFI) and posterior tibiofibular interval (PTFI), which determine the anterior tibiofibular ratio (ATFR). All measurements are calculated 1 cm above the the centre of the tibial plafond and their positive reliability has been described in many studies [8,9]. The MCS on the AP view of the ankle, should be less than 4 mm and the TFO should be more than 6 mm [7], while on a true lateral view, the ATFR needs to be 40% (approximately 40% of the tibia should lie anteriorly to the anterior fibula cortex).

## Materials and methods

**Figure 1:**
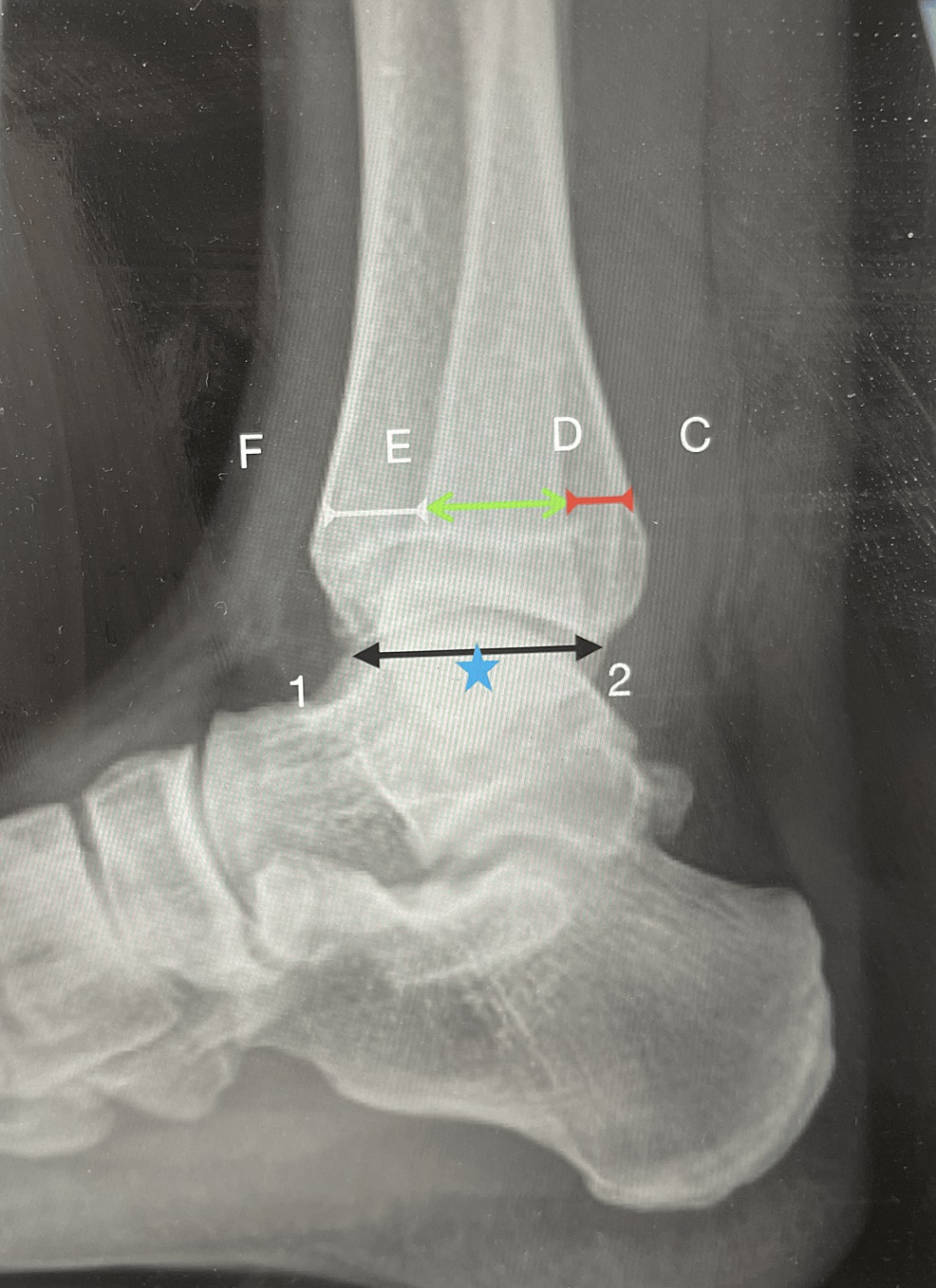
Example of radiographic analysis F-E indicates the anterior tibiofibular interval, E-D indicates the fibular width, D-C shows the posterior tibiofibular interval, 1-2 indicates the tibial plafond, * indicates the midpoint; all measurements were performed 1 cm above the midpoint.

This was a retrospective study aiming to evaluate the radiographic performance of two different surgical techniques in the management of ankle fractures including syndesmotic disruption. The analysis was conducted on 93 patients of all ages who underwent an ankle fixation procedure in a single institution in the UK (Luton and Dunstable University Hospital) between January 2021 and April 2022, with a minimum radiographic follow-up of one year. All operations were performed by two different trauma surgeons and the implant used was based on surgeon's preference. Depending on the implant used, all cases were divided into two groups: TightRope system group (Arthrex, Naples, FL) and syndesmotic screw fixation group. Radiographic evaluation of the postoperative radiographs was performed (Figure 1).

**Figure 2:**
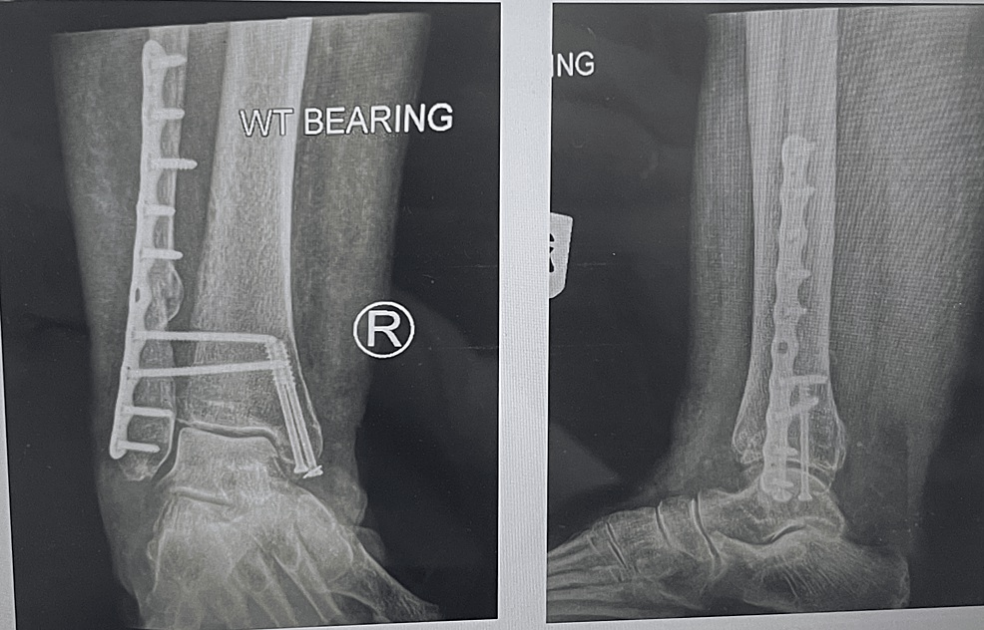
Postoperative radiograph of the bimalleolar ankle fracture with syndesmotic disruption, addressed using syndesmotic screws

**Figure 3:**
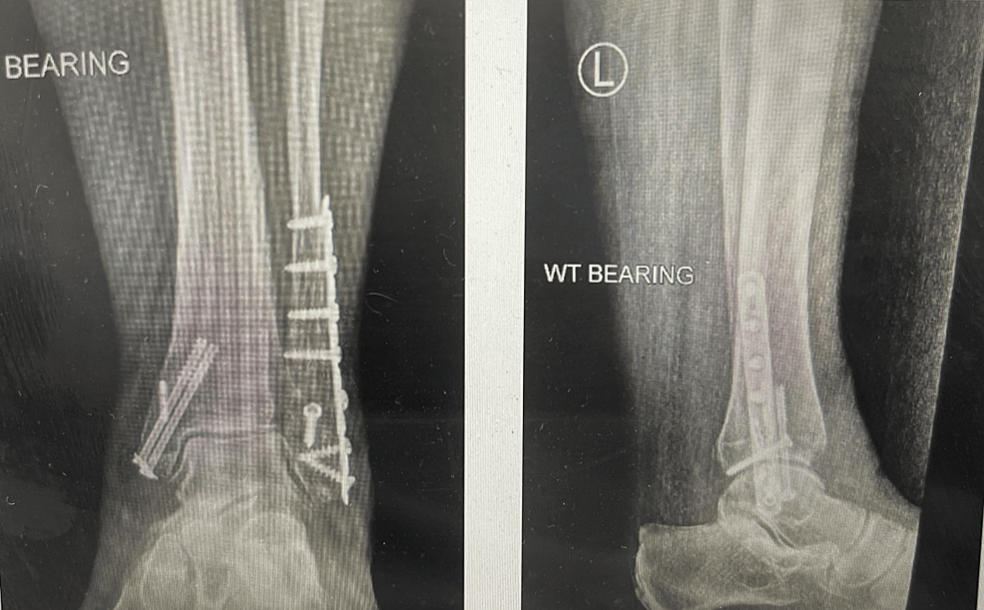
Postoperative radiograph of the bimalleolar ankle fracture with syndesmotic disruption stabilised using the TightRope system

Only cases of ankle fractures with an underlying syndesmotic injury were included in the study (as defined from the preoperative planning) and the cases were assigned to two groups, depending on the type of fixation they received at the time of the procedure. Patients in the syndesmotic screw fixation group were treated using syndesmotic screw fixation and the TightRope group included patients who received TightRope system stabilisation for the underlying syndesmotic injury (Figures 2, 3). The choice of the implant used was solely based on surgeon's preference. Patient demographics, type of ankle fracture (isolated fibula fracture, bimalleolar or trimalleolar ankle fracture) and type of implant used (syndesmotic screw vs. TightRope system) were included in the statistical analysis. Exclusion criteria included open ankle fractures, revision procedures, combination of implants (syndesmotic screw and TightRope system) and a radiographic follow-up of less than a year. The radiographic analysis was performed by four independent reviewers and cases with no consensus among the reviewers were excluded from the study.

For the syndesmotic screw fixation group, out of the 54 cases initially identified, six cases were excluded following the exclusion criteria (four cases due to inadequate postoperative radiographs and two cases with inadequate follow-up, less than a year) and 48 cases were included, whereas for the Tightrope system group, out of the 39 cases originally identified, two cases did not meet the inclusion criteria and were excluded (one case due to inadequate postoperative radiographs and one case with a follow-up of only three months). In total, 48 cases for the syndesmotic screw group and 37 cases for the TightRope group were included in the study.

The primary aim of this study was to assess if there was any statistical significant difference in the radiographic syndesmotic restoration between the two groups as seen on the postoperative static radiographs; the secondary aim was to assess if the postoperative anterior tibiofibular ratio varied between the two different surgical techniques. For all cases, both dynamic and static postoperative radiographs were obtained, but the statistical analysis was based on the static views, in order to reduce the risk of bias as the interpretation of dynamic postoperative radiographs can be challenging, with many different unpredictable factors affecting the outcome (weight bearing status, position of the foot, ankle positioning in the neutral position, etc.). We employed statistical analysis (chi-square test) for both groups and the results were assessed by all the reviewers.

## Results

In the syndesmotic screw fixation group (48 cases), 19 cases were classified as trimalleolar ankle fractures (in four cases, the posterior malleolar fragment stabilised with AP screws, and in five cases, with a posterior plate, with the rest of the cases not receiving any type of fixation of the posterior malleolar fragment), 12 cases as bimalleolar injuries (all treated with fibula plate fixation and partial threaded screw fixation for the medial malleolar fragment with two screws) and 17 cases as isolated fibula fractures with syndesmotic disruption (Table 1). In the TightRope system group (37 cases), 14 cases were identified as trimalleolar injuries (three cases received AP screws for the posterior malleolar fragment, and two cases a posterior plate, with the rest of the cases not receiving any type of posterior malleolar fixation), 8 cases as bimalleolar (all treated with standard fibula plate fixation and medial malleolus fixation using two partial threaded screws) and 15 cases as isolated fibula fractures with syndesmosis instability (Table 2). The overall mean age of the 85 patients included in the study was 53.4 years, and there were 47 females and 38 males (Table 3).

**Table 1:** Radiographic evaluation of the syndesmotic screw fixation group MCS, medial clear joint space; TFO, tibiofibular overlap EF indicates the anterior tibiofibular interval and CD is the posterior tibiofibular interval. Data has been represented as mean±SD.

Syndesmotic screw group	Number of cases	Mean MCS	Mean TFO	Mean EF	Mean CD
Bimalleolar fractures	12	3.2±0.4	7.7±2.1	12.9±2.7	6.1±2.8
Trimalleolar fractures	19	3.1±0.6	7.4±1.9	12.4±3.3	5.8±2.7
Fibula fractures with taller swift	17	2.9±0.5	7.6±2.5	11.8±2.9	6.4±3.1
Total	48				

**Table 2:** Radiographic evaluation of the TightRope stabilisation group MCS, medial clear joint space; TFO, tibiofibular overlap EF indicates the anterior tibiofibular interval and CD is the posterior tibiofibular interval. Data has been represented as mean±SD.

TightRope stabilisation group	Number of cases	Mean MCS	Mean TFO	Mean EF	Mean CD
Bimalleolar fractures	8	3.1±0.5	7.8±1.8	12.7±2.4	5.9±2.9
Trimalleolar fractures	14	3.3±0.4	7.3±2.1	12.1±3.1	5.8±2.8
Fibula fractures with taller swift	15	3.2±0.3	7.5±1.9	11.7±2.6	6.2±2.9
Total	37				

**Table 3:** Demographic data F, female; M, male

	Syndesmotic screw fixation group	TightRope system stabilisation group
Gender	26 F/22 M	21 F/16 M
Mean age	54.3 years	52.1 years
Total number of patients	48	37

For all the 85 cases included in the study, radiographic evaluation was performed on the AP, mortise and true lateral view of the postoperative radiographs at six weeks and three months after the initial procedure, while during the one-year follow-up review, the postoperative radiograph was also assessed to confirm radiographic congruency of the ankle joint prior to discharge. For the syndesmotic screw fixation group, six cases (12.5%) were identified as having a medial clear joint space of more than 4 mm (4.7-6.6 mm) during the six-week postoperative AP radiograph, versus three cases (8.1%, p=0.42) in the TightRope system group, during the same postoperative period.

The tibiofibular overlap was assessed on the postoperative AP view for all the patients; six cases (12.5%) were identified in the syndesmotic screw group with TFO measuring less than 6 mm (4.6-6 mm) versus three cases (8.1%) in the TightRope group.

The radiographic evaluation of the true lateral view of the ankle included the assessment of the anterior and posterior tibiofibular interval, 1 cm above the midpoint of the tibial plafond in order to determine the anterior tibiofibular ratio for all cases. Nine cases (18.75%) were found with a postoperative anterior tibiofibular ratio of less than 40% in the syndesmotic screw fixation group and three cases (8.1%, p=0.16) in the TightRope system group. No statistical correlation was noted with regard to the type of ankle fracture treated, as all three different types of ankle injuries (isolated fibula ankle fractures, trimalleolar ankle fractures and bimalleolar ankle fractures) had a similar distribution of radiographic abnormal parameters during the postoperative analysis (Pearson correlation -0.014, -0.094 and -0.074, respectively).

## Discussion

The importance of syndesmotic stability has been well described in several studies [10,11], leading to detrimental effects for the patients when disrupted and left untreated [12,13]. Diagnostic arthroscopy, CT and MRI scans have been introduced as a safe and effective way of diagnosing syndesmotic injuries [9]. However, recent financial pressures on national health systems and long waiting times for a radiographic examination (CT, MRI scans) make the need to diagnose and treat syndesmotic injuries adequately via careful clinical examination and adequate orthogonal radiograph interpretation more important than ever [7]. Traditionally, the syndesmotic injuries have been treated using a syndesmotic screw, with many different variations available, in terms of the number of screws used, cortices used, screw size, placement of the screw, the use of a washer with the screw and the use of a screw through a plate [14].

The use of TightRope is a new alternative solution, which offers a more dynamic rather than static way of syndesmosis stabilisation [15]. The evidence of its use is mixed, but a recent randomized controlled trial (RCT) demonstrated that TightRope use was linked with a well-maintained reduction in the syndesmosis after two years whereas the use of trans-syndesmotic screw was associated with a slight increase in malreduction rates after two years of follow-up [16]. Several studies indicate that the TightRope system provides an effective way of syndesmosis stabilisation, avoiding the need for implant removal providing a dynamic rather than a static restoration of the syndesmosis [17,18]. As a result, the use of the TightRope system avoids several of the limitations of the traditional syndesmotic screw fixation technique [19]. Common complications of the syndesmotic screw fixation technique such as breakage of the metalwork [20], loosening of the screw [21], synostosis of the distal tibiofibular space [22], over-reduction of the syndesmosis [23] and restrictions in the dorsiflexion of the ankle [23] can be avoided by introducing a suture fixation system through the tibia and fibula [24], allowing not only stabilisation but also normal range of movement.

In terms of clinical outcomes, the effectiveness of the TightRope system has been well described [24]. The use of the TightRope system seems to provide clinical and patient-reported outcomes similar to the conventional syndesmotic screw fixation but with quicker recovery time and return to activity [17,24].

In line with the above-mentioned RCT, our study shows that both surgical techniques (TightRope system and syndesmotic screw fixation) seem to provide similar radiographic outcomes in terms of radiographic analysis of syndesmosis stability, with no statistical significant difference detected between them, but at the same time, the overall rate of malreduction of the syndesmosis (as indicated by the MCS, TFO and ATFR) in the TightRope group seems to be lower in comparison to the syndesmotic screw group. This could be possibly explained by the more rigid and static way of syndesmosis stabilisation that the syndesmotic screw provides in comparison to the more dynamic and flexible mode of fixation of the TightRope system. In our case series, the radiographic evaluation of the postoperative radiographs did not reveal any cases of syndesmosis over-reduction (over-compression), which is a well-described complication following the management of ankle fractures with syndesmotic disruption [23,24]. Possibly this could be attributed to the fact that all operations were performed by two experienced trauma surgeons, and despite the use of intra-operative reduction clamp for all cases, serial intra-operative radiographs were obtained for all the cases showing adequate anatomic restoration of the tibiofibular interval.

Among the limitations of the study were the relatively short minimum follow-up period of one year, its retrospective character and the inclusion of different types of ankle fracture patterns presenting with syndesmotic instability (isolated fibula fractures, bimalleolar and trimalleolar ankle fractures). None of the patients from the syndesmotic screw group had the syndesmotic screw(s) removed during the one-year follow-up. Another limitation could be the fact that all procedures were performed by experienced trauma surgeons and not dedicated foot and ankle specialists, but for all cases included in the study, a preoperative multidisciplinary orthopaedic meeting was conducted in the presence of a specialist foot and ankle surgeon to determine the optimal surgical plan. Also, the radiographic evaluation of the syndesmotic congruity was based on the analysis of orthogonal radiographs rather than the interpretation of CT scans. Patient satisfaction rate, complication rate and functional assessment scores were not included in this study, as these were considered to be beyond the primary aim of our investigation. To our knowledge though, this is among the few studies focusing on a true radiographic evaluation of ankle syndesmotic injuries by comparing two different surgical techniques, with an adequate number of cases.

## Conclusions

At a time of increasing financial pressure on national health systems and the increasing patient waiting time for an advanced radiographic investigation (CT, MRI scans), the need for accurate assessment of syndesmosis stabilisation based on orthogonal radiographs is becoming crucial. In our study, the methodical and careful interpretation of the postoperative radiographs along with a thorough clinical examination of the patients seemed to be adequate in assessing the stability of the syndesmosis postoperatively, in patients undergoing an ankle fracture procedure.

Despite no statistically significant differences in the radiographic parameters of the postoperative radiographs between the TightRope system group and the syndesmotic screw fixation group, a lower incidence of radiographic incongruity for the TightRope group was noted postoperatively. Providing a more dynamic mode of stabilisation of the syndesmosis, the TightRope system is gaining popularity in the management of ankle fractures with syndesmotic instability. In comparison to the traditional syndesmotic screw fixation technique, which requires a second intervention for metalwork removal and also carries the risk of breaking of the screw during rehabilitation, the TightRope system seems to provide an effective way for the management of ankle syndesmotic injuries.
